# Ti(C,N) and WC-Based Cermets: A Review of Synthesis, Properties and Applications in Additive Manufacturing

**DOI:** 10.3390/ma14226786

**Published:** 2021-11-10

**Authors:** Lida Heydari, Pablo F. Lietor, Francisco A. Corpas-Iglesias, Oscar H. Laguna

**Affiliations:** Higher Polytechnic School of Linares, University of Jaén, Av. de la Universidad s/n, 23700 Linares, Jaén, Spain; pflietor@ujaen.es (P.F.L.); facorpas@ujaen.es (F.A.C.-I.); olaguna@ujaen.es (O.H.L.)

**Keywords:** carbides, cermets, composites, additive manufacturing, 3D printing

## Abstract

In recent years, the use of cermets has shown significant growth in the industry due to their interesting features that combine properties of metals and ceramics, and there are different possible types of cermets, depending on their composition. This review focuses on cemented tungsten carbides (WC), and tungsten carbonitrides (WCN), and it is intended to analyze the relationship between chemical composition and processing techniques of these materials, which results in their particular microstructural and mechanical properties. Moreover, the use of cermets as a printing material in additive manufacturing or 3D printing processes has recently emerged as one of the scenarios with the greatest projection, considering that they manufacture parts with greater versatility, lower manufacturing costs, lower raw material expenditure and with advanced designs. Therefore, this review compiled and analyzed scientific papers devoted to the synthesis, properties and uses of cermets of TiC and WC in additive manufacturing processes reported thus far.

## 1. Introduction

Cemented carbides are composites with excellent properties; thus, they have attracted attention in various scientific fields. These composites exhibit combined properties of carbides and metal binders, with the concurrent presence of toughness and hardness. There are generally three different types of cemented carbides: cemented carbides based on tungsten carbide with cobalt binder phase, tungsten carbide in combination with TaC and NbC, and TiC with cobalt binder phase and cermets [1]. Generally, two groups of tungsten-based carbide (WC) have been made for years, with cobalt (WC-Co)- or nickel (WC-Ni)-based binders [2]. Cemented carbides show different properties, depending on the processing steps and their components, which distinguishes them in applications. For example, WC-Co has a high hardness due to the wettability of tungsten by cobalt, which is used for cutting tools. Conversely, addition carbides such as titanium carbide (TiC), tantalum carbide (TaC) and niobium carbide (NbC) to carbide tungsten with cobalt binder improve corrosion resistance of straight WC-Co grades [3,4,5,6,7]. Compared with TiC-based cermets, TiCN-based cermets have much higher high-temperature hardness, higher transverse rupture toughness, better resistance to oxidation, and much higher thermal conductivity [8].

The conventional methods for fabrication of cermets are associated with excess consumption of precursors and the use of complex equipment; in addition, it is impossible to access advanced geometric structures; thus, use of an additive manufacturing technique has largely removed these obstacles. Additive manufacturing (AM) technologies are defined as the “process of joining materials to make objects from three-dimensional (3D) model data, usually layer by layer, as opposed to subtractive manufacturing and formative manufacturing methodologies” [9,10]. AM technologies include three-dimensional printing (3D printing), which is equivalent to inkjet printers, and uses materials, instead of ink, to build three-dimensional objects. Several 3D printers have been invented since the late 1970s [11,12]. The main difference between all these processes is in how layers are deposited and the starting materials used [13,14].

**Scheme 1:**
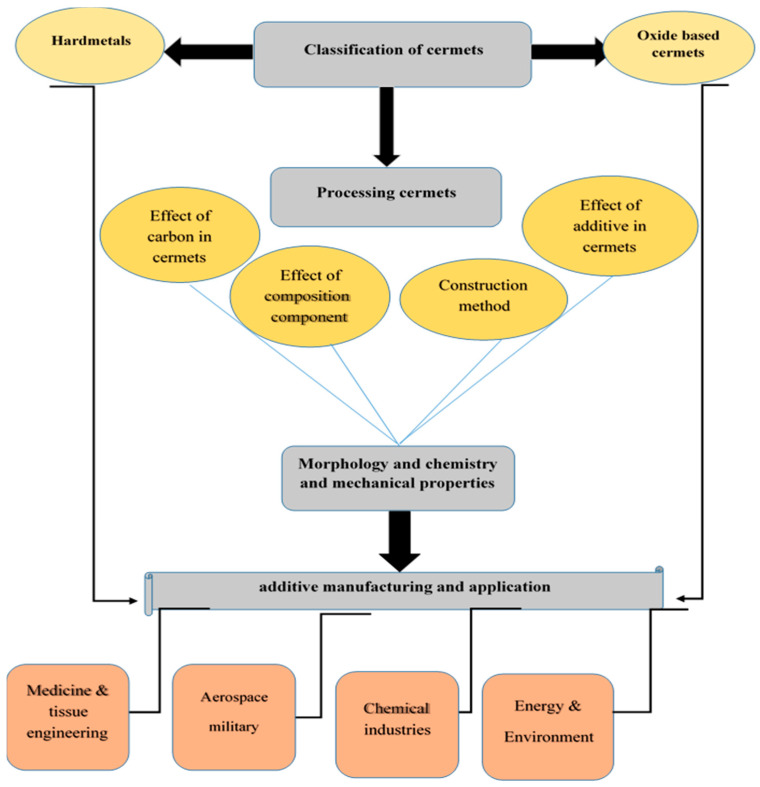
Flow diagram of aspects evaluated in the article.

The purpose of this work is to classify ceramic–metal composites and their processing. Here, we try to explain additive manufacturing for the fabrication of elegant 3D objects in geometrical complexity and size with high performance. All aspects and purposes of the work are presented in the flow diagram (Scheme 1).

## 2. Definition, Classification and Milestones in the History of the Cermets

**Scheme 2:**
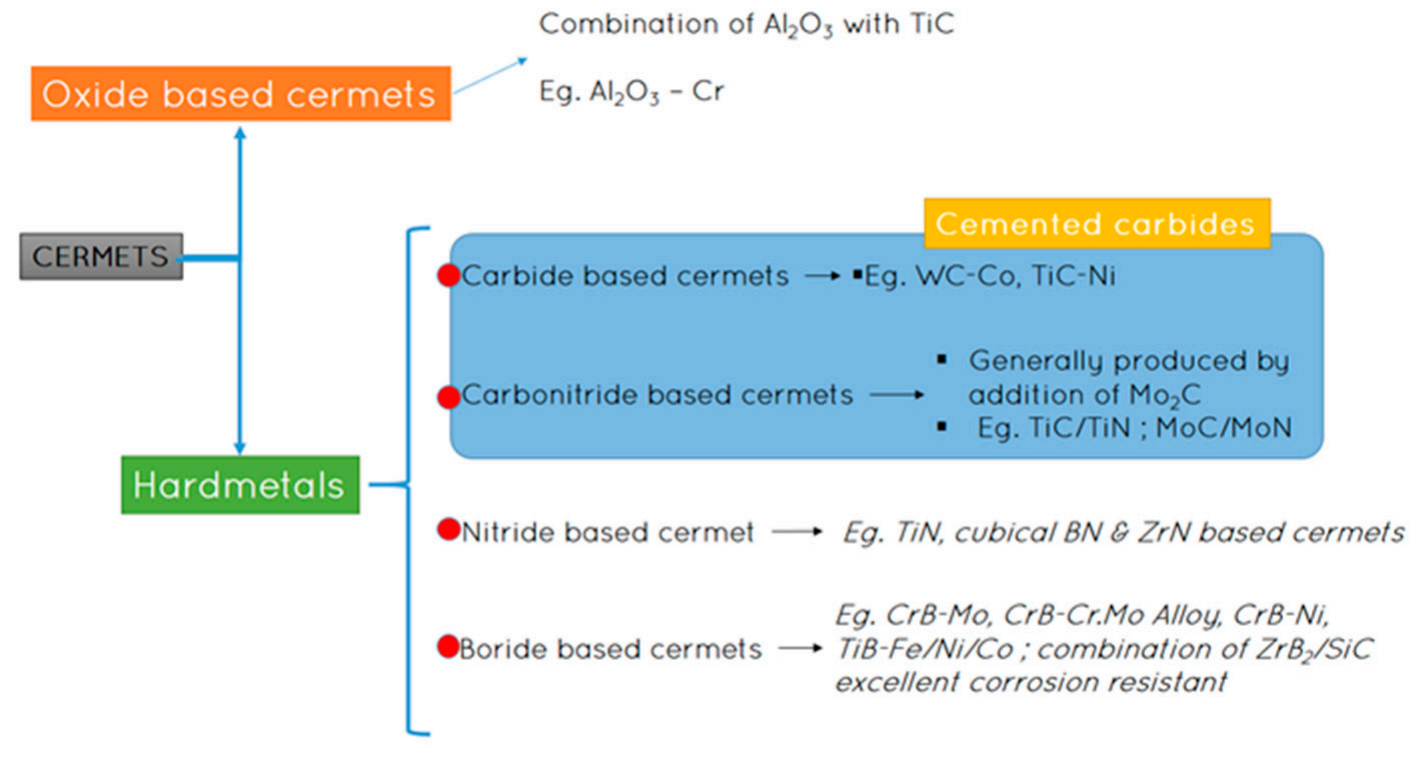
Classification of cermets.

Cermets include a ceramic matrix bonded by a metallic binder [12]. Two prominent groups of cermets can be defined: hard metals, which are based on carbides, and oxide cermets. The oxide cermets are shown in the schematic below (Scheme 2).

**Figure 1:**
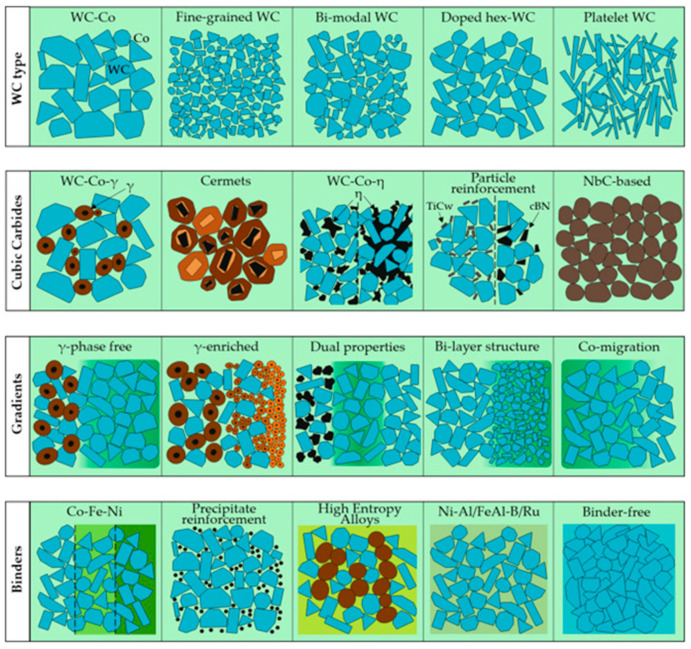
Categorization of cemented carbide microstructures. Reprinted from Ref. [18] with permission from Int. J. Refract. Met. Hard Mater.

In cemented carbides, the hexagonal tungsten carbide forms the dominant phase [15]. There are two phases in cemented carbide: (1) tungsten carbide, which is called alpha-phase (α), and (2) the cobalt binder, also called beta-phase (β). Additionally, the third component of cubic carbides and carbonitrides appear to be new properties, as they act similar to tungsten carbide grain growth inhibitor. This phase is called the gamma-phase (γ) (Figure 1) [16]. The most widely used cermet consists of tungsten carbide (WC) with about 6% cobalt [2]. The TiWCN-Co cermet has a core–rim structure, where the cores are pure cubic TiCN and the rims are TiWCN, which will be discussed later, regarding their morphology [17].

Presented in Figure 1 is the classification of microstructures of cemented carbides and cermets.

Cemented carbide was developed by Osram Lamp Works in 1923 and introduced in the market by Krupp AG in Germany in 1927 as a cutting tool under the tradename of “WIDIA” [19]. The first cermets, containing titanium carbide/molybdenum carbide with nickel as a binder were manufactured in Germany in 1929 [20]. This class of materials also contains WC or Mo_2_C to improve the wetting of the hard phase and increase mechanical performance. During the 1950s, cutting tool manufacturers offered a new TiC–nickel alloy cermet that contained molybdenum and carbon [21]. Another improvement of cermet properties came in 1973, from adding titanium nitride (TiN) [22]. Increasing research had been conducted until industrial products were no longer marketed by 1980, and Japan played a major role in the production of TiCN-based cermets until 1990 [23]. A new group of ternary carbides with aluminum has been studied since 2006, which has great potential in the field of automotive and aerospace applications [24,25]. A comparison between carbon tool steel and carbides steel have shown that carbon tool steel, including carbon and alloy steels, decrease in hardness with increasing temperature, and this limits the carbon tool steel for cutting tools. Conversely, high speed steel is a high carbon tool steel. High speed steel compensates the above-mentioned defects and is used to manufacture complex thin blades. However, carbide steel, such as tungsten carbide, has a high red hardness, including at 1000 °C, where it still has a high hardness [26,27,28,29]. Parashivamuthy et al. reviewed various reinforcements and processing routs in titanium-reinforced steel matrix composites [28]. Carbon nanotubes (CNTs) are candidates for reinforcing multifunctional composites, but there is poor interfacial bonding between CNTs and the matrix. Coatings of CNT with carbides improve interfacial properties. The formation of titanium carbide on the surface of CNT and its effect on the properties of Al composites was studied. In optimal conditions, there was observed an increment in yield strength for Al-modified CNTs (84.36 MPa) compared with pure Al (54.49 MPa), Al-CNT (65.64 MPa) [30].

## 3. Procedures for Obtaining Cermets

**Figure 2:**
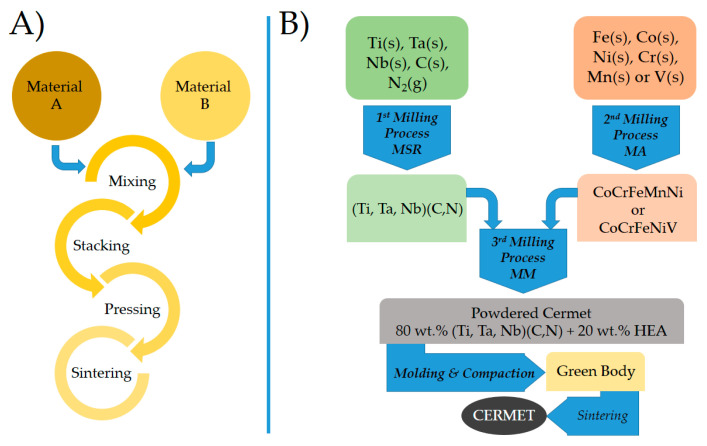
Steps in the synthesis of cermets: (**A**): traditional method, (**B**): advanced method. Adapted from Refs. [26,31].

There are traditional and advanced methods for the synthesis of carbide cermets. Figure 2 shows a flow diagram of two methods for the synthesis of cemented carbides that explain some of carbide specimens by various processing along effective parameters in the fabrication process [26,31].

**Figure 3:**
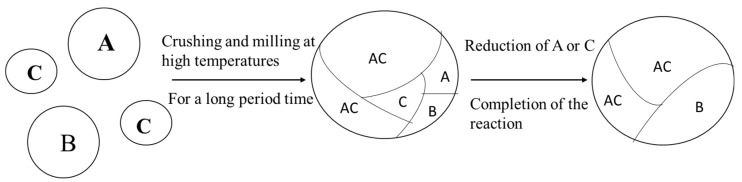
Traditional carbide cermet synthesis processing. Reprinted from Ref. [32] with permission from Mater. Sci. Eng.

Traditional methods for obtaining cemented carbides involve mechanical mixing and high temperature reactions, such as the synthesis of chromium carbide cermets. The steps are shown in Figure 3.

Traditionally, cemented carbides are produced by liquid phase sintering processes, which often lead to undesirable growth of tungsten carbide grains, even with the addition of grain growth inhibitors such as VC, Cr_3_C_2_ [33]. In WC-Co cemented carbides, WC grain size seriously affects the mechanical properties. Some scientists have solved this problem through chemical methods. Kear and Kim, in 1990, described an aqueous solution reaction (ASR) method for producing nanostructured WC-Co, with an average WC grain size of 30–50 nm [34,35]. Regarding the sintering step for preparation of cemented carbides, a higher temperature (than for hard metals) is required, and novel techniques have been proposed to reduce grain size and lower sintering temperatures to produce fully dense materials [36,37]. Formation of mesostructures can be achieved through high technologies of sintering that apply hot isostatic pressing (HIP), spark plasma sintering (SPS), high frequency induction heated sintering (HFIHS), rapid omni compaction (ROC), pulse plasma sintering (PPS), and ultrahigh pressure rapid hot consolidation (UPRC) [38]. Lagos et al. reported the fabrication of chromium carbide cermets by electric resistance sintering process. Chromium carbide cermets prepared by this novel method have higher strength properties than those prepared using the traditional method. Cr_3_C_2_-25NiCr (wt.%) and WC-20Cr_3_C_2_-7Ni (wt.%) are suitable for use in abrasive and corrosive environments [39]. In addition, they reported the development of the electrical resistance sintering (ERS) process for fabrication of hard metals WC with 6 and 10 wt.% of Co, and the results were compared with conventional processes [40]. Potschke et al. reported synthesis of dense (Hf, Ta, Nb, Ti, V)C- and (Ta, Nb, Ti, V, W)C-based high-entropy carbides (HEC) using three different sintering techniques: gas pressure sintering/sinter–HIP, vacuum sintering, and SPS/FAST [41]. Zhao et al. prepared ultrafine-grained WC-Co bulk materials by a new method that includes pretreatment of the milled powder mixture and subsequent spark plasma sintering (SPS) [42]. SPS is known as a popular method because it obtains a uniaxial pressure and a rapid heating cycle to consolidate materials. SPS creates a short sintering time at high temperature; subsequently, particle size control is provided. In addition, unwanted reactions between WC and cobalt are minimized [43]. In the SPS process, three inclusive factors (current, pulse, and discharge) influence interactions between powder components and the formation of different phases [44]. Zhong et al. prepared WCoB-based cermets by spark plasma sintering at a sintering temperature range of 600–1200 °C. The maximum value Vickers hardness was 1751 Hv0.5 at 9.356% Cr doping content [45]. Ghasali et al. fabricated WC-based cermets using Ni and Mo as the binder phase through spark plasma sintering. WC/Ni cermet showed a higher bending strength (1260 ± 48 MPa) and fracture toughness (17.27 ± 1.14 MPa m^1/2^) in comparison with WC/Mo (bending strength and fracture toughness of 455 ± 29 MPa and 8.77 ± 0.61 MPa m^1/2^, respectively) because of the good wetting behavior of the Ni binder used [46].

**Figure 4:**
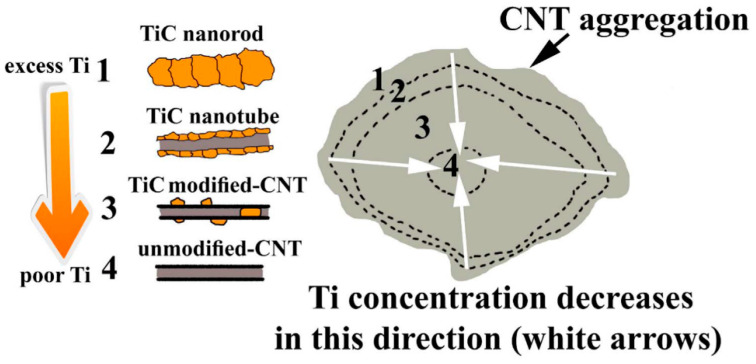
Schematic diagram of the formation mechanism of TiC nanorods, nanotubes and TiC-decorated CNTs. Reprinted from Ref. [55] with permission from Ceram. Int.

Yung et al. reported synthesis of ZrC-20 wt.% Mo cermet by spark plasma sintering at various temperatures ranging between 1600 and 2100 °C under either 50 or 100 MPa of compaction pressure. This particular sintering regime gave 396 GPa Young’s modulus due to small clusters of Mo across the microstructure [47]. Breval et al. investigated and compared microwave and conventional sintering of WC/Co composites. Microwave sintering was recommended due to low energy consumption. The microwave sintered samples had 1–5 GPa better hardness compared to the conventionally sintered samples [48]. This can be attributed to the nature of the heat transfer mechanisms involved. The direct reduction and carburization process was considered one of the best methods to prepare nano-sized WC powder. Lee et al. reported synthesis of WC-5–10 wt.% Co by the carburization method, and the mean size of WC/Co composite powder was about 260 nm [49]. Ban and Show used a novel route known as the “integrated and mechanical and thermal activation” (IMTA) to synthesize nanostructured WC-Co powder. The properties of the resulting nanostructures powder are analogous to those of nanostructure powders prepared using conventional preparation methods [50]. The direct reduction and carburization are well known in achieving fine microstructure particles without agglomeration, which is also used in the preparation of TiC composites [51]. Wang et al. used nanosized SiC particles as reinforcement in the production of TiC by spark plasma sintering (SPS). It was observed to be fully dense and included improvements in the fracture toughness of the composite, arising from the SiC addition as an inhibitor of the coalescence of TiC grains [52]. Among the ceramic–metal composite materials, titanium carbonitride and titanium carbides, as basic materials, are equivalent to tungsten carbide for application as a cutting tool. TiC is an interesting ceramic material because of its hardness, strength, wear resistance, and other properties. Ding et al. reported fabrication of TiC cermet by high-energy ball-milling with Ti, granular activated carbon, and Ni powders via spark plasma sintering (SPS) treatment [53]. Zhang et al. proposed pressureless SPS for the production of porous nano-ceramic scaffolds in 2008. Saba et al. modified nanocrystalline TiC particles onto the surface of multiwall carbon nanotubes by using pressureless SPS. This strategy is valuable, as it is possible to produce a variety of nano-carbide coated carbon nanotubes, carbon nanofibers, and graphene that are difficult to produce with other methods [54]. CNTs have been used as a template to prepare many kinds of nanocarbides, such as TiC, NbC, SiC nanostructures. Saba et al. presented a formation mechanism of a TiC-modified carbon nanotube during pressureless SPS at 1050 °C (Figure 4). The morphology of produced nanomaterials strongly depends on Ti concentration from the periphery to the center of CNT clusters [55].

**Figure 5:**
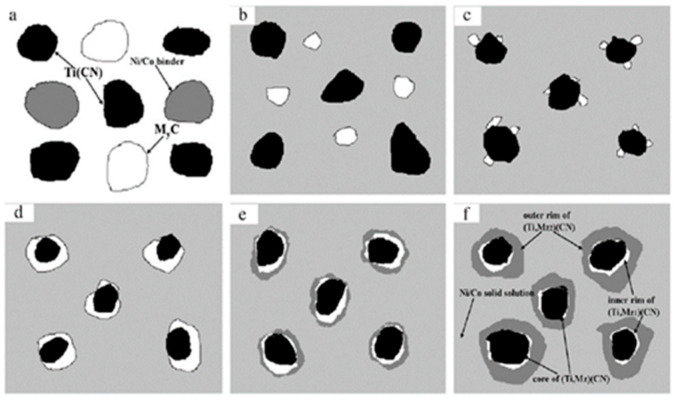
The illustration of structure characteristics in Ti(C,N)–MXC cermets (**a**) 20 °C; (**b**) 950 °C; (**c**) 1150 °C; (**d**) 1300 °C; (**e**) 1350 °C; (**f**) 1450 °C. Reprinted from Ref. [57] with permission from Int. J. Refract. Met. Hard Mater.

Ti(C,N)-based cermet presents a core/rim structure by liquid phase reactions. These structures undergo many variations during certain conditions. The cores consist of partly undissolved raw Ti(C,N) particles, onto which (Ti,W)(C,N) rims grow through a dissolution–reprecipitation mechanism during sintering. Monteverde et al. synthesized fine Ti(C,N) powders. This powder was mixed with 15.3 wt.% of WC(Co) + 6.2 wt.% of Ni + 2.1 wt.% of Co and was hot pressed [56]. Li et al. synthesized Ti(C,N)-based cermets from Ti(C,N)\WC\Mo_2_C\TaC\Ni\Co composite powders by vacuum low-pressure sintering. The evolution path of core–rim is described by means of illustration in Figure 5 [57].

Yan et al. constructed Ti(C,N)-based cermets using β-Co microspheres. Cermets were prepared from β-Co as the binder phase and use a (Ti, W, Mo, Ta) (C, N) solid instead of single metal carbide. Variation precursors are accompanied by interesting variety structures. Two kinds of core–rim structures appeared to possess fine cores and thick rims [58]. Precursors, sintering, temperature processing, atmosphere processing and binders are important factors for grain growth. For example, increasing nitrogen amounts to titanium carbonitride (TiCN) as an added precursor creates uniform structure and small grain size. This path provides more stability by obtaining the same structure core and shell [59]. Zhao et al. synthesized Ti(C,N)-based cermet with different TaC/(TaC + WC) weight ratios by in-situ reactive hot pressing. The cermets contained three kinds of core–ring structure grains: black core–gray ring, dark gray core–gray ring, and dark gray core–white inner ring with a gray outer ring. As the TaC/(TaC + WC) weight ratio increases, because of Ta, it has a higher tendency to promote the formation of (W, Ta, Ti) (C, N) solid solution; thus, the thickness of the ring phase gradually increases [60].

Yu et al. fabricated TiC-TiB_2_-based cermets with Ni binder using combustion synthesis assisted by pseudohot isostatic pressing through heating the compacted powder mixture to approximately 700 °C. TiC-TiB_2_-Ni (52.5/17.5/30) exhibited high relative density of 93.45% and high hardness of 1950 HV [61].

Table 1 shows the sintering techniques for the preparation of cermets with different binders.

### 3.1. Effect of Carbon in Cermets

It was found that the carbon content of alloy can be adjusted through the addition of carbon or tungsten carbide. To achieve the excellent properties of the WC–Co bulk, a combination of initial powders having the appropriate amount of carbon is essential [77]. The sintering atmosphere affects carbon content of cermets and subsequently the microstructures and properties.

**Figure 6:**
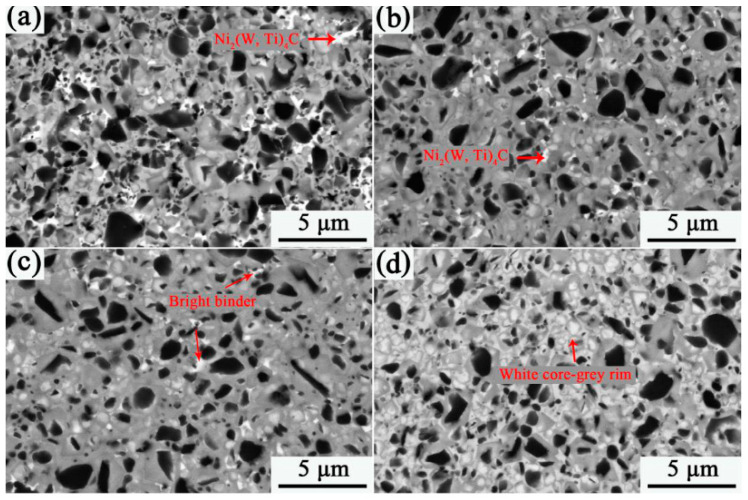
SEM images of V0.5 to V1.5 sintered in gas pressure sintering at 1470 °C for 1 h with 0.5 and 0.8 wt.% C. (**a**) V0.5; (**b**) V0.8; (**c**) V1.2; (**d**) V1.5. Reprinted from Ref. [83] with permission from Ceram. Int.

Kear et al. investigated carburization treatments of WC-Co reduced powders in CO/CO_2_, CO/Ar and CO/H_2_ gas mixtures. It was established that a CO/H_2_ mixture provid-ed better control of the formation of uncombined or free carbon, while maintaining a high carburization rate [34]. Kim et al. observed that WC grains in WC–Co material exhibits various forms, and that the deformation is reversibly related to C content. Increasing car-bon content from 0.1% to 1.0% C indicated that WC grains tend to show an elongated rec-tangular form [78]. Formisano et al. reported the influence of eta-phase on wear behavior of WC-Co carbides. The wear resistance of WC-Co carbides containing Eta-phase was an-alyzed. The presence of a secondary phase in the hard metals as an eta-phase occurs when carbon is deficient [79]. Conversely, Graphite is formed when the carbon content is too high [80]. Eso et al. proved that the η phase formed in the samples is Co3W3C [81]. There is a critical value carbon content that can be avoided from eta-phase or precipitation of graphite. Besharatloo et al. showed that the addition of C reduced porosity in all cases and enhanced hardness. This C addition effect was more pronounced when the binder content is increased [71]. Zhang et al. reported that the micromorphology of the core–shell structure in the cermets was strongly affected by carbon content. Increments of carbon content can lead to a finer hard-core phase and thicker rim phase in the cermets [82]. Xiong et al. showed that average grain size and thickness of the rim phase in Ti(C,N)-based cermets increased with higher carbon content or gas pressure sintering, alt-hough this event is derived from the accelerated dissolution–precipitation process [83]. Figure 6 shows that, in high carbon content (1.2 or 1.5 wt.%), small particles will evolve into white core–grey rim phases or grains with thicker inner rims. Conversely, at high cooling rates, dissolved Ti, W, Mo, C and N atoms have a short time to separate out. Hence these atoms may be retained in binder, leading to a bright phase.

Liu et al. studied the microstructure of Ti(C,N)-based cermets in different carbon contents. Increment carbon content produced the finer grain size. The η-phase present in the low carbon cermets was known as (Ni_2_Mo_2.7_W_1.1_) C_x_ intermetallic [84].

Zhaoa et al. prepared a composition of TiC-10TiN-32Ni-16Mo-(9-x) WC-xC (x = 0, 0.5, 1.0 and 1.5 wt.%) by vacuum sintering. The carbon addition had a significant effect on cermets sintered at 1400 °C. The dissolution process of carbides in Ni binder reduced with higher carbon amounts appeared to show core and rim structures with a thin rim [85]. The investigation of (Ti,W) (C,N)-Co cermets composed of black core/grey rim and white core/grey rim grain revealed fractions of undissolved Ti(C,N) cores declined with increments of carbon. It was followed by an increase in an amount of white core/grey structure [83].

### 3.2. Effects of Additives in Cermets

In cermets, the main part is the matrix phase, and the minor part is the binding phase. The interactions between these parts determine the final structure. Therefore, the grain size and content of the binder are sufficient to explain the structures and properties. It is important to control the growth of WC grains, which is achieved by using grain growth inhibitors. The addition of grain growth inhibitors is also effective in improving fracture toughness [84]. Chermant and Osterstock studied the correlation between strength parameters and volume fraction of cobalt and between grain sizes of tungsten carbide [86]. Chen et al. reported the additional amount of VC in Ti-C based cermets. VC mainly dispensed around the outer rims, if it increased, and the black cores dissolved, while the average thickness of the rims increased slowly. Cermets with 4 wt.% showed the best mechanical properties, with a bending strength of 1028 Mpa and a Vickers hardness of 1640 Nmm^−2^ [87]. Siwak et al. produced WC–Co cermets using the addition of Cr_3_C_2_ and TaC grain growth inhibitors via spark plasma sintering, but Cr_3_C_2_ had better grain growth inhibition than TaC [88]. Farag et al. rated the effect of different dopants aspect grain inhibition potential as follows: VC > NbC > TaC/TiC>Mo_2_C/Cr_3_C_2_ > ZrC/HfC (valid at low doping amounts) [89].

**Figure 7:**
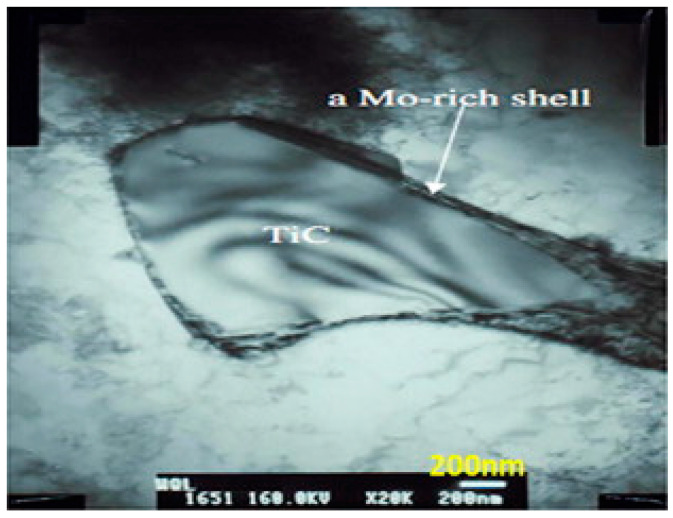
TEM bright field image showing a Mo-rich shell around TiC in Al-alloy (Al–0.6Cu–0.6 Mg–1.2Si–0.25Mn–10TiC–1Mo). Reprinted from Ref. [92] with permission from Chem. Eng. J.

Nino et al. reported the addition of TaC and TiC on the microstructure to improve grain growth [84]. The addition of TaC to WC ceramics produced the solid solutions of (W, Ta)_2_C and (Ta, W)C as reaction products. A small TaC improved mechanical properties of WC. Li et al. prepared a titanium matrix composite (TMCs) that was reinforced with TiC particles and TiB whiskers via powder metallurgy and hot extrusion. The synthesized TMCs with a 13.6% volume fraction of TiC–TiB hybrid reinforcements presented ultimate tensile strength of 1138 MPa, representing a 74% improvement compared to pure titanium [90]. Kear et al. studied the use of VC as a grain growth inhibitor for the preparation of WC-Co through a new thermochemical processing method [34]. Generally, VC is an effective additive to limit grain growth in the solid state because of the formation of a thin film (V,W)C_x_ on the surface of WC [74]. Konyashina et al. prepared WC-Co-Re cemented carbides. Rhenium acts as a strong WC grain growth inhibitor, suppressing the WC coarsening process [91]. Mo, as an additive in TiC-based cermets, shows two approaches: (i) increasing the wettability binder and hard phase ceramic (Figure 7), and (ii) reducing the solubility of TiC or Ti(C,N) in binder, which leads to restricted grain growth. Defective wetting is known as a factor of creation coagulation of the carbide phase and presents coarse particles. Therefore, mentioned approaches of Mo can improve the mechanical behavior of TiC-based cermets [92].

Zhang et al. reported that Mo can enhance the corrosion resistance of WC–TiC–Ni hard metals. This may be a reason for the formation of new (Ti, W, Mo)C phase based on TiC [93]. In recent years, other additives such as MgO, CaO, TiO_2_, La_2_O_3_, SiO_2_, TiC, TiB_2_, ZrB_2_, AlN, and TiN have been introduced to further improve the mechanical, physical properties and the microstructure of SiC ceramics [94]. Modified CNT by nanocrystalline TiC is well known as a reinforcement in Al nanocomposites. In the optimum value of the mixing ratio TiC:CNT, CNT weight fraction exhibited 176% and 71% increase in Vickers hardness, compared to the pure Al and unmodified-CNT/Al composite sample, respectively [95].

In choosing the type of additive, possible reactions between initial materials should be considered. Olubambi et al. showed that the addition of zirconia to 9Co/WC–4.5CO–2Cr–5Ni reduced hardness that was related to grain growth; notwithstanding, toughness improved with an increase in zirconia content. It can be said that the mechanism sintering changed in the presence of metallic and zirconia binders. [75]. Kimmari et al. prepared WC-based cermets with the addition of ZrO_2_ in three mixtures through a sinter/HIP routine, and conditions were defined as: presence of cobalt as binder, presence of nickel as binder, and the last one in the absence of binder [96]. The porosity in the mixture with cobalt (86 wt.% WC, 8 wt.% Co and 6 wt.% ZrO_2_) as well as in the case of mixture without metal (86 wt.% WC and 14 wt.% ZrO_2_) was higher, and it may be described through poor wettability of Co-ZrO_2_ or ZrO_2_ in WC.

## 4. Morphology and Chemistry

**Figure 8:**
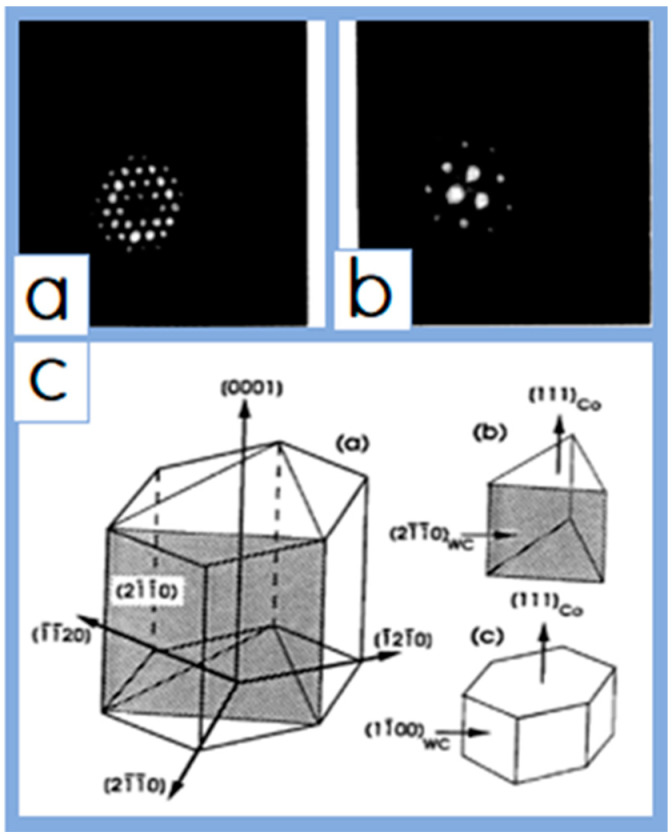
Electron diffraction analysis for nanostructured WC-6%Co-0.8%VC following 8 hr heat-treatment at 700 °C. Micro–micro diffraction patterns for (**a**) (0001) zone axis of matrix, (**b**) (111) zone axis of fee Co nanoprecipitate. (**c**) (a) the Co nanoprecipitate morphology with reference to the hexagonal WC unit cell, (b) and (c) the triangular and hexagonal forms, as deduced from the electronic microscopy observations. Reprinted from Ref. [99] with permission from Nanostruct. Mater.

Tungsten and carbon are available in two forms of different carbides: WC and W_2_C. The phase mostly present in cermets (hard metals) is monocarbide. It can be said that more than 98% of all hard metals contain WC and more than half of them are pure WC-Co. WC grains have a hexagonal crystal structure with two atoms per unit cell and a c/a ratio of 0.976 when the interfaces are assumed to be coherent [97,98]. The W-C system implies peaks of (001) and (100) of tungsten monocarbide α-WC and peaks of (100), (002), and (101) of tungsten semi-carbide αW_2_C. Figure 8a,b shows the (0001) micro–micro diffraction pattern of the hexagonal WC phase. A hexagonal-form shape in the (0001) projection corresponds to an ultrafine Co precipitate with interfaces parallel to the (1100) planes of the WC matrix phase [99].

This kind of additive and carbon content influence the morphology and mechanical properties of cermets. Delanoë et al. evaluated the effect of Cr addition and of C content on the shape of WC-Co. The addition of Cr increases the anisotropy between prismatic facets in tungsten-rich alloy [100]. Yuan et al. reported correlation of mechanical properties and interface area aspect ratio derived from the relative area of prevalent (0001) basal planes and (10-10) prismatic planes [101]. Lay et al. showed that the grains became less truncated and flatter in C rich alloys of WC–(24 wt.%) Co [102]. 

Ti(C,N) has a common structure, that is, a sodium chloride structure, wherein carbon atoms on the TiC super-lattice can be replaced by nitrogen atoms [103]. The structure of Ti(C,N) core/rim is formed from dissolution of Mo or Cr into Ti(C,N), in which some of the Ti atoms were substituted by other metal atoms [104].

Three sections including: core, inner rim and outer rim undergo variations that depend on the atmosphere of sintering and variety of powder components. The core was surrounded by the inner rim due to diffusion of W, Mo and other atoms during solid sintering. However, the outer rim was an identical solution, similar to the inner rim, but with less W, Mo and other heavy elements originating from the dissolution–precipitation through liquid sintering [4]. Chao et al. reported two kinds of core/rim structures in Ti(C,N) cermets. The use of TiC as an initial powder resulted in a black core/grey rim that varied depending on whether fine or coarse titanium carbide powder was used. The thickness of the outer rim was smaller, while the inner rim structure was blurred when fine TiC powder was used. Conversely, coarse TiC powder was well dissolved in the liquid binder phase, and subsequently, the thicker outer rim was formed. A new kind of core/rim structure (white core and grey rim) was produced using Ti(C,N) as a starting powder. Because of the extremely small particle size of Ti(C,N), the diffusion length between Ti(C,N) core and precipitated rim was significantly reduced. As a result, large amounts of relatively small Ti(C,N) cores were completely consumed by the growing inner rim. As the sintering temperature increased, the thickness of the outer rim increased, and these structures were dominated along with improved mechanical properties [105,106].

The magnetic methods are efficient to control the mean size of the grains of tungsten carbide and carbon content in the cobalt interlayer after sintering. Since some of pure cobalt in cermets will be combined with carbon or tungsten, consequently, normal magnetic properties of pure cobalt changes. Co alloyed with WC and Co alloyed with W_2_C (Eta phase) are not magnetic [107]. Another magnetic parameter that is used for measurement quality of cermets is the coercive force (Hc). Coercivity is defined as magnetizing a sample (cemented carbides) in a strong magnetic field. Regarding mentioned points about magnetic cermets, it can be said that coercivity is a measure of how well cemented carbides have been sintered because, during sintering, cobalt can produce non-magnetic alloys. Conversely, it has a direct relationship with grain size, as finer-grain WC shows higher coercivity [16]. Manuel et al. studied the effect of high energy milling for the composite WC-10%wt Co. There is a significant increase in the coercive field and a decrease in the saturation magnetization when milling time increases [108]. Xu et al. synthesized Ti(C0.6, N0.4)-8Mo-xWC-25Ni (x = 0, 3, 6 and 9 wt.%) cermets under different cooling rates by vacuum sintering. The Cermets became paramagnetic at x = 6 under the cooling rate of 2 °C/min; x = 6 and 9 under the cooling rate 35 °C/min [109].

## 5. Cermets for Additive Manufacturing and Application

Traditional manufacturing methods to fabricate cermets are limited in various paths, such as choice of starting material, control of sintering parameters, and difficulty of access to different shapes and structures. Additive manufacturing includes the processing steps of: (a) digital model of the object; (b) facet model; (c) cross-sectional data by 3D printers. As a result, it is possible to prepare objects with geometric complexity, compared to conventional methods [110]. The most common 3D printing methods include: fused deposition modeling (FDM), stereolithography (SLA), selective laser sintering (SLS), and robocasting or direct ink writing (DIW) [111]. Powder-bed fusion techniques such as selective laser sintering (SLS), selective laser melting (SLM), and electron beam melting (EBM) use powder material [112]. The research has extensively covered additive manufacturing of WC-Co hard metals that are produced by powder metallurgy, including conventional liquid-phase sintering [113]. Fries et al. processed WC with 17 wt.% Co by laser powder-bed fusion (LPBF) with a powder-bed heating of 900 °C [114].

Konyashin et al. obtained dense WC-Co by a single-step based on SEBM without post sintering in Sinter-HIP furnaces. These carbides were non-porous, but due to the growth of local grains, abnormal large grains appeared to be a challenge [115]. Cramer et al. synthesized WC-Co composites using binder jet additive manufacturing (BJAM), following melted metal binder Co. The composites had less shrinkage and grain growth compared to other techniques [116]. Ravi et al. prepared WC-12% Co via binder jet 3D printing. The printed samples were sintered under a pressure of 1.83 MPa at 1485 °C for 5 min to achieve near theoretical densities. The wear resistance of BJ3DP carbides was excellent [117]. The first research on the laser sintering of tungsten carbide-cobalt was carried out at the University of Leuven in Belgium [118]. Cemented WC materials are ideal for SLM processing due to the lower melting temperature of the binder phase compared to the melting temperature of WC (2870 °C) [119]. Khmyrov et al. changed the WC/Co ratio to study cracking. They processed the powder mixture with 25 wt.% WC by SLM to obtain materials without crack. The powder mixture with 50 wt.% WC cracked because of formation of the W_3_Co_3_C phase [120]. Kumar et al. optimized parameters of SLS for preparation of WC-Co. They showed that higher laser power gave rise to cracks and depletion of cobalt, while higher scan speed increased porosity [121]. Ku produced samples of WC with 10 wt.% Fe-Ni-Zr binder using SLM [122]. Other transition metal binders had been used as binders due to the harmful effects of cobalt on humans. Gu et al. showed that densification of TiC/Ti nanocomposite parts using the SLM method was affected through laser energy density and powder categories [123]. The experiments revealed that a fully dense TiC/Inconel 718 part was fabricated at a proper E of 300 J/m because of nano-TiC reinforcement, which experienced severe agglomeration to the uniform distribution along the grain boundaries and inside the grains of the matrix [124]. Aramian et al. showed that TiC-NiCr cermets fabricated by selective laser melting using maximum energy density had the highest mean microhardness [125]. Deckard et al. invented selective laser sintering (SLS) in 1986, which used powders as starting materials in this 3D printing preparation approach [123]. Kumar produced a composition of WC–9 wt.% Co by SLS machine, and bronze infiltration was performed on laser-sintered parts to enhance wear resistance for cutting tools [126]. Lu et al. milled Cu–Ti–C and Cu–Ni–Ti–C powder mixtures, and then powders were layered and scanned under the laser beam (SLS). The addition of Ni improved the microstructure and surface quality of the laser-fabricated parts because of the betterment melting of Cu and wettability between Cu and TiC [127]. Grigoriev et al. reported wear-resistant WC-Co made by selective laser melting (SLM). The optimal conditions were 94 wt.% WC and 6 wt.% Co with 2500 HV [128].

The material extrusion methods, defined as an “additive manufacturing process in which material is selectively dispensed through a nozzle or orifice’’, and it contains two subgroups: fused deposition modeling and fused filament fabrication [129]. Shakor et al. reported that extrusion printing is a fluent technique that can be adapted for rapid and arbitrary construction projects [130]. This technique includes useful subsets such as robocasting, robotic deposition, and direct ink writing. In general, it can be said that hard metal and cermet were printed by fused-filament fabrication (FFF) in an SDS (shaping–debinding–sintering) process. Filaments were prepared from hard metal (WC-10Co) and cermet powder (Ti(C,N)-Co/Ni-based) and organic binder [129]. Three-dimensional gel-printing (3DGP) is a novel manufacturing technology that makes a 3D component by depositing and gelating metal slurry layer by layer. This method removed the barriers to allow the use of high strength polymer gels in the industry. Zhang et al. made the hydroxyethyl methacrylate (HEMA)-based slurries with WC-20Co solid loading of 47–56 vol.% by 3DGP, which was sintered in a vacuum furnace. The printed samples had good accuracy in term of complexity in shapes and homogeneity in the microstructure [131]. The principle of stereolithography (SLA) is based on a photopolymerization process and typically exploits some kind of light source to cause the chemical reaction in monomers and oligomers to cross-link together [132]. The SLA/DLP application in the mold, healthcare and jewelry industries is becoming the fastest growing market [125]. Varghese et al. applied digital light processing (DLP) and projector UV source to fabricate green bodies from photosensitive resins loaded with 25–60 wt.% of alumina, 3- and 8-YSZ. The 3D-printed bodies were sintered in the 1200–1500 °C and exhibited thermal stability [133]. Sun et al. produced ZTA printing material through DLP. The materials of ZTA ceramic slurry inclusive powders and UV resin with various ZrO_2_ concentrations (5–15 wt.%) were investigated, and their rheological behavior was tested to determine 3D printing performance [134].

Liu et al. reported a specific capacitance of 391 F/g at 1 A/g for the PANI micro-structures as an electrode in a supercapacitor that was prepared using PDMS molds by SLA. They consumed polymer resin as a template (indirectly produced structure). Furthermore, laser interference lithography was employed to fabricate nano-structures on the micro-structures. The specific capacitance of 487 F/g was obtained [135]. Afonso et al. presented the use of SLA-3D printing technology to produce customized YSZ microstructures [136]. Hernandez-Rodriguez et al. fabricated 3D ceramic microstructures based on the yttrium-stabilized zirconia (YSZ) by SLA methods. The microstructure ceramic components were stable after sintering at 1400 °C for 4 h [137].

Liu et al. manufactured ZrO_2_-Al_2_O_3_ composite ceramic parts by SLA-3D printing and conducted a subsequent debinding and sintering process. The hardness and fracture toughness of the ceramics were examined through optimized maximum sintering temperature and holding time [138]. Chen et al. mixed hydroxyapatite powder into photosensitive resin to form a complex shape scaffold using SLA-3D printing technology [139]. In SLA-3D printing technology, light scattering is increased if the fillers and resin have large differences in their refractive indexes [140]. SLA is differentiated from the most common fused deposition modeling (FDM), not only by utilization of resin, but also because of the unbelievable resolution that the technology enables.

Cemented tungsten carbide (WC) is widely used for wear-resistant applications, such as cutting tools and abrasives [141]. Cemented carbide (CC) is a useful material for mining tools [142]. WC-Co nanoparticles have been introduced as another option for use in the fields of cosmetics, biomedicine, electronics, etc. [143]. However, the mechanical properties of WC-Co are influenced by quality WC powders. The hardness of the cemented carbide with WC powders, owing to high crystallinity, decreases slightly, while the fracture toughness and transverse rupture strength increases [144]. Using YSZ as an additive, in series of WC–6Co cemented carbides produced a good microstructure along excellent mechanical properties [76]. Acchar et al. produced WC-Co reinforced with NbC by hot-pressing in an inert atmosphere. The WC-Co-NbC composite material exhibited high hardness values (18.9 GPa), flexural strength (2100 MPa) and fracture toughness (11.2 MPa m^1/2^) when used as cutting tool materials [145]. An example of the specifications and applications of the cermets is suggested in Table 2.

Despite the advantages, however, there have been concerns about using Co as binder in cermets, because of the toxicity of Co [146]. There are several studies based on using Fe as a metal binder for cermets, as it is non-toxic and cheaper than other metals, while it confers more strength to the final structure after post-thermal treatments [147]. TiC-based cermets are superior to WC-based hard metals in some cases, such as lower friction coefficients and higher oxidation resistance [148]. Cermets based on TiC bonded with Fe-alloy (steel) binder showed preference over TiC base composites bonded with Ni alloy in all wear conditions [149]. Cermets, usually based on titanium carbide (TiC) or titanium carbonitride (TiC_x_N_y_), have excellent wear and corrosion properties [150]. Therefore, combining the electrical conductivity of the metal binder and the stability of titanium carbides can be considered a suitable option for SOFC. Xie investigated the stability of TiC-Ni and TiC-Ni_3_Al cermets for SOFC-interconnected application [151]. Qi proposed promising metal matrix composites (TiC/Ni–Cr) for SOFC-interconnected applications [152].

**Figure 9:**
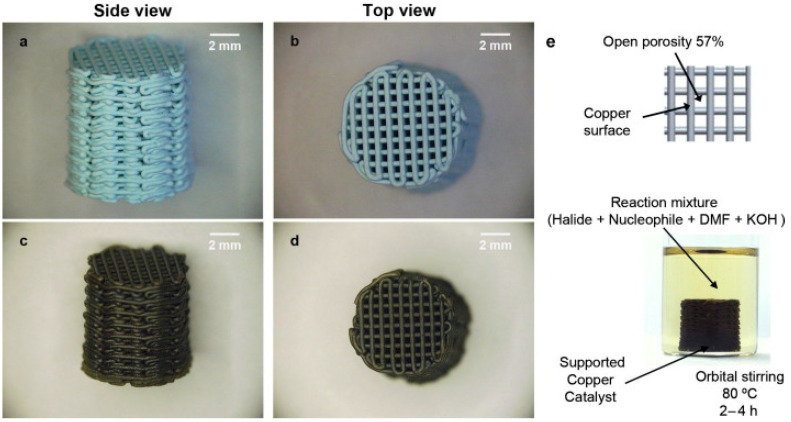
Optical images of the Cu/Al_2_O_3_ structure: dried (**a**,**b**) and sintered (**c**,**d**). Schematic illustration and image of the experimental set used for catalytic tests (**e**). Reprinted from Ref. [157] with permission from J. Biomed. Mater. Res.

TiN has a number of advantages over common cemented carbides, including smaller grain size, higher wear resistance, and thermodynamic stability [153]. Gai et al. reported that novel TiN-Ni cermets can be used as promising interconnections for practical application of IT-SOFCs [154]. Cardinal et al. prepared cermets based on TiC and TiN with Ni as binder. The effect of TiN addition and binder content on the microstructure and the properties of the TiC based cermets were investigated for applications as tools [155]. Ceramics are formed using conventional technologies, including injection molding, die pressing, tape casting, gel casting, etc., but they have barriers to access arbitrary and complex objects. It was found that the preparation using traditional fabrication methods such as casting and machining is difficult [156]. Additive manufacturing offers a smother route to achieve geometric flexible heterogeneous catalysts or especially catalyst support production. Nevertheless, 3D printing of functional materials as a catalyst defines new challenges, including a restriction on material selection, process adaptions, thermal post-processing steps, and catalytic testing [110]. Tubío et al. printed a reusable copper catalyst system using robocasting of a Cu/Al_2_O_3_ slurry. The woodpile-like structure was catalytically active for the Ullman reaction (Figure 9) [157]. Diaz-Marta et al. demonstrated the compartmentalization of Cu and Pd catalytic sites in two different 3D printed SiO_2_ monoliths [158].

PtO_2_-WO_3_ catalysts with complex shapes were manufactured via digital light processing. The 3D catalysts were tested for the hydrogenation of alkynes and nitrobenzene and showed high activity in these catalytic reactions [159]. Wei and co-workers showed that metal 3D printing products can simultaneously act as chemical reactors and catalysts (self-catalytic reactor SCR). Fe-SCR and Co-SCR catalyze Fischer-Tropsch synthesis and CO_2_ hydrogenation; Ni-SCR efficiently produces syngas (CO/H_2_) by CO_2_ reforming of CH_4_ [160]. Today, the conversion and use of CO_2_ is an attractive and promising solution to reduce greenhouse gas emissions [161]. Laguna et al. reported graph diagrams that correlate to different printing techniques, raw materials, type of catalytic reaction ware, and reactions in which devices are used in most cases [162].

Cruz et al. provided a description of the required different steps for manufacturing a microchannel reactor for the CO-PROX reaction [163]. Laguna reviewed effective parameters of structured catalysts (monoliths and microreactors) such as metal choice and method of deposition of the catalytic layer on the substrate [164]. The monoliths catalysts can display hierarchical structure by combining intrinsic active materials and interconnected channels to facilitate mass and heat transfer. These structures possess high surface area to load a more catalytic active phase, ultimately increasing performance.

Afonso employed the binder jetting technique to produce ceramic support of CaSO_4_, which acts as support to the catalyst for the removal of contaminants from wastewater [165].

AM techniques can be used for fabrication of ceramic membranes, owing to enhanced selectivity and productivity. For example, prepared membranes from metals, alloys, and ceramics by SLM are applicable in water treatment [166]. In addition, the application of 3D printing and digital modeling (CAD, CAM) for the design of chemical reactors for advanced oxidation processes (AOPs), in particular photo-fenton reactors, has been investigated [167]. Dijk and co-workers have recently presented an optimization analysis of external light trapping, with the fabrication of 3D-printed external light traps in forms of square, hexagonal, and circular compound parabolic concentrator arrays [168]. Biswas prepared clay-based honeycomb substrates showing channel structures of square, hexagonal, and triangular shape by 3D printing and evaluated as a reactor in treating sewage. Macro-channels and microchannels were completely connected through structure engineering for increment reaction rates [169]. Tripathi and Behera reviewed concepts of honeycomb structural materials, engineering design and manufacturing; 3D-woven honeycomb composites have wonderful properties for extensive applications [170]. Lyu fabricated the 3D surface-patterned alumina membrane with porous structures through a layer-by-layer coating of the fabricated ceramic ink onto the porous substrate [171].

**Figure 10:**
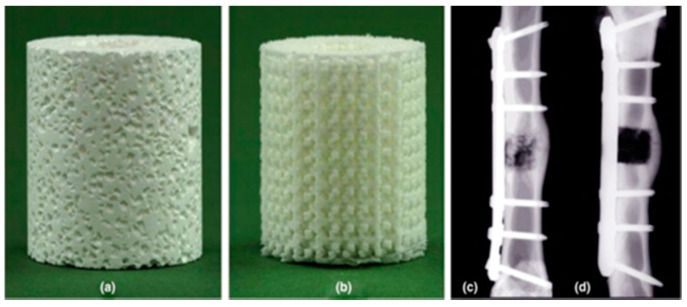
Commercial β-TCP implant (**a**) and PCL/TCP scaffolds built by SLS (**b**). X-ray images of tibia defects treated with β-TCP (**c**) and PCL/TCP (**d**) scaffolds after 14 weeks of implantation. Reprinted from Ref. [176] with permission from Acta Biomater.

Sun et al. prepared two configurations of alumina (Al_2_O_3_) ceramics (hollow lattice structure and solid lattice structure) by DLP 3D printing technology that can be used in industrial thermal insulation applications [172]. Mora et al. presented CNT/polylactic acid (PLA) and CNT/high density polyethylene (HDPE) composites via fused filament fabrication (FFF). The combined properties of filler nanoparticles and conducting polymer through 3D printing were multiplied. Electro-conductive polymer nanocomposites (PNCs) have been widely used for applications such as interference shielding, flexible electronics, electrodes and batteries [173]. Gnanasekaran et al. constructed 3D-printed PBT/CNT composites by FDM along superior mechanical properties [174]. Three-dimensional printing of scaffolds is called bioceramic scaffolds, and they are designed to mimic complex tissue such as songs, fibrous, gradient, and woven. Biomaterials used for scaffolds include metals, ceramics, glass-ceramic, polymers and composites. Three-dimensional scaffold structures with highly controlled micro/macro porous and excellent mechanical properties are widely used in biomedical applications. Therefore, properties of scaffolds are analyzed based on electrical conductivity, mechanical and chemical surface, and topology arising from material interests and selection of production technique. Iron and nickel scaffolds are used in energy storage, emission control, catalyst support, and structural applications [175]. The three-dimensional-printing products have been applied to tissue engineering. Lohfeld et al. evaluated in vivo performance of a commercial β-TCP (tricalcium phosphate) implant and a composite PCL/TCP scaffold produced by SLS (Figure 10a,b) [176].

Simon et al. reported excellent results for bone growth using hydroxyapatite (HA) scaffolds fabricated by direct ink writing (DIW) with 3D periodic pore architectures [177], while other authors have presented a successful example of an occlusal surface of a dental crown produced through a material jetting process, from a ceramic suspension of yttria partially stabilized zirconia [178,179]. Cermet cements, due to their ease of preparation and placement, cariostatic effect, and adhesion to tooth substance, have some advantages over commonly used materials. Glass-ionomer/cermet is used in building up cores on posterior teeth. Glasses containing fluoride were among the earliest reported that include either the SiO_2_-Al_2_O_3_-CaF_2_ system or the more complex SiO_2_-Al_2_O_3_-P_2_O_5_-CaO-CaF_2_ system [180]. 

## 6. Conclusions and Outlook

In this review, the various types of cermets and hard metals were discussed. Combining the properties of ceramics and metals with a binder yields materials with new properties. Parameters such as grain size and chemical composition components can be a good indicator of their mechanical properties. Providing optimal conditions in the starting materials, additives and sintering conditions leads to desirable morphology. The mechanism of sintering and consolidation is responsible for cemented carbide microstructures. WC-Co composites have outstanding mechanical properties that are influenced by microstructures. A uniform distribution of metal phase in ceramics improves mechanical properties. In sintering, there occurs a reduction surface area which results in densification of green compact. Conventional sintering processes have limitations such as long heating step and coursing of nanoparticles. Novel sintering techniques appeared to minimize grain growth and enhance densification. Ti(C,N)-based cermets have been employed in high-speed finishing operations because of good high temperature hardness, superior thermal conductivity, excellent creep resistance, etc. The microstructure of Ti(C,N)-based cermets exhibit a “core–rim” structure, which is controlled by dissolution and reprecipitation mechanisms. Average grain size and thickness of rim phase are affected by carbon content and sintering. The difference in material properties between metal and ceramic can make stress singularities at the interface deteriorate the strength of the ceramic–metal joint. Conversely, difficulties in adequate dispersion powder and control of metal concentration are challenges and opportunities for more investigations in fabrication cermets. However, improving the processing of cermets is an effective step for economic production. Conventional processes such as powder metallurgy techniques and casting showed limitations. Additive manufacturing (AM) has emerged as a useful method to produce porous ceramic parts with complex geometries. AM-based techniques have been used for the fabrication of different metallic, polymers, ceramics and cermets. Chemical composition and different process parameters have been evaluated by researchers for achievement in AM cermet. Although successful fabrication of cermets with desirable qualities was reported by AM processes, this technique suffers from difficulties for fabrication-dense parts. Obtaining a proper feedstock formulation for fabrication of full dense ceramic with optimal properties is the main problem. The preparation of cermets with higher density and suitable morphology requires post-processing such as heat treatment, separating component from support structure, hot isostatic pressing, etc. on AM cermets. However, the solution to challenge the preparation of cermets by using AM techniques requires extensive research. 

However, there is no doubt that production of ceramics and cermets through AM will be improved with close participation from academia and the industry.

## Figures and Tables

**Table 1:** Sintering techniques for preparation of cermets.

Composition (wt.%)	Sintering Techniques	Hardness Vickers	Mean Grain Size µm	Ref
Cr_3_C_2_-25NiCr (wt.%) WC-20Cr_3_C_2_-7Ni (wt.%).	Electric Resistance Sintering (ERS)	1060 ± 42 1110 ± 30 (HV30)	-	[39]
WC6Co	ERS	1960 ± 15	-	[40]
	Sinter-HIP	1860 ± 15		
	ERS	1750 ± 20		
WC10Co	Sinter-HIP	1620 ± 15 (HV30)		
(Hf, Ta, Nb, Ti, V)C (Ta, Nb, Ti, V, W)C	Sinter-HIP Vacuum SPS Sinter-HIP Vacuum SPS	2437 ± 47 2573 ± 86 2569 ± 56 1873 ± 80 2217 ± 40 2021 ± 56 (HV_0.5_)	2.59 ± 0.16 3.46 ± 0.18 5.62 ± 0.38 15.2 ± 0.82 33.7 ± 1.80 31.2 ± 1.81	[41]
WC-Co (Ultrafine WC)	SPS	1836 (HV30)	0.28	[42]
ZrC-20%Mo	SPS	2231 (HV10)	1–2	[47]
Ti(C,N)-TiB2-WCTaC-Co	Hot pressing sintering	2052 (HV10)		[60]
TiC-TiB_2_-Ni(52.5/17.5/30 vol.%).	HIP	1950 (HV50)		[61]
WC-Co (9–10 wt.% Co) WC-7 wt.% Co, 0.3wt.% CNT	SPS SPS	- 2450 (HV30)	0.4–0.8	[62]
WCoB	SPS	1262 (HV30)	-	[63]
WCoB-9.356wt.%Cr	SPS	1751 (HV_0.5_)	-	[63]
cBN-WC-Co	SPS	2170 (HV30)	-	[64]
WC–5Co–2Cr_3_C_2_ WC–5Co–2TaC	SPS SPS	2105 ± 38 1725 ± 15 (HV30)		[65]
WC-Ni-ZrO_2_	HIP	1600 (HV10)	3–5	[66]
WC–12Co-1%Cr_3_C_2_ WC–12Co	SPS HP	1872 1668 (HV30)	-	[67]
(Ti(C,N))–WC–Mo–Ni/Ni + Co–C	Vacuum sintering	1756 ± 52 (HV100)	0.64 ± 0.3	[68]
Ti(C,N)/Mo-Co-Ni/CaF_2_@Al_2_O_3_	Vacuum sintering	2440 (HV30)		[69]
(NbC-15 vol.% Mo)-12 vol.% Ni	Vacuum sintering	1466 (HV30)		[70]
T70/FeCr20Mn20	Vacuum sintering	1450 (HV30)		[71]
WC–7.5 wt.% NanoCo	SPS	1925 (HV30)	1–5	[72]
WC-12Co-1VC	SPS	2000 (HV30)	-	[73]
WC-11 Co, 0.5 wt.%VC	HIP	1902 (HV30)	-	[74]
WC–4.5CO–2Cr–5Ni	SPS	2000 (HV30)		[75]
WC-Co-9wt.%YSZ	SPS	1520 (HV30)		[76]

**Table 2:** Characteristics of hard metal and cermet grades and their application [141].

Composition	Grain Size (µm)	(wt.%)	Application
WC-Co	Nano < 0.2	8–12	PCB drills, punches for ink jet printer heads, microtools
WC-Co	Ultrafine 0.2–0.5	2–4 6–9 10–16	Wood cutting tools, wear parts Microdrills for PCB, cutting inserts Rotating tools, paper knifes
WC-Co	Submicrometer 0.5–0.8	4–16	Cutting inserts, rotating tools
WC-Co	Fine 0.8–1.3	4–25	Cutting inserts, wear parts Noncutting shaping tools
WC-Co	Medium 1.3–2.5	4–25	Cutting inserts for heavy cutting Noncutting shaping tools
WC-Co	Coarse 2.5–6.0	4–25	Rock cutting tools
WC-Co	Extra coarse >6.0	4–25	Forming tools
WC-Co	0.5–2	4–20	Chemical apparatus engineering Corrosion resistant nanomagnetic parts
WC-Co	0.5–2	4–15	Cutting tools for steel chipping
Cermets	0.5–3	5–20	Cutting tools for steel chipping

## Data Availability

Not applicable.
